# Pulmonary Hypertension Associated Genetic Variants in Sarcoidosis Associated Pulmonary Hypertension

**DOI:** 10.3390/diagnostics12102564

**Published:** 2022-10-21

**Authors:** Karlijn Groen, Marloes P. Huitema, Joanne J. van der Vis, Marco C. Post, Jan C. Grutters, Robert P. Baughman, Coline H. M. van Moorsel

**Affiliations:** 1 Department of Pulmonology, St. Antonius ILD Center of Excellence, St. Antonius Hospital, Koekoekslaan 1, 3435 CM Nieuwegein, The Netherlands; 2 Department of Cardiology, St. Antonius Hospital Nieuwegein, Koekoekslaan 1, 3435 CM Nieuwegein, The Netherlands; 3 Department of Clinical Chemistry, St. Antonius ILD Center of Excellence, St. Antonius Hospital, Koekoekslaan 1, 3435 CM Nieuwegein, The Netherlands; 4 Department of Cardiology, University Medical Center Utrecht, 3584 CX Utrecht, The Netherlands; 5 Division of Hearts and Lungs, University Medical Center Utrecht, 3584 CX Utrecht, The Netherlands; 6 Department of Medicine, University of Cincinnati Medical Center, Cincinnati, OH 45219, USA

**Keywords:** pulmonary hypertension, sarcoidosis, genetic variants, BMPR2, NOTCH3

## Abstract

Background: Pulmonary hypertension (PH) is a severe complication of sarcoidosis in a minority of patients. Several genetic defects are known to cause hereditary or sporadic PH, but whether variants in PH-associated genes are also involved in sarcoidosis-associated PH (SAPH) is unknown. Methods: 40 patients with SAPH were individually matched to 40 sarcoidosis patients without PH (SA). Whole exome sequencing was performed to identify rare genetic variants in a diagnostic PH gene panel of 13 genes. Additionally, an exploratory analysis was performed to search for other genes of interest. From 572 genes biologically involved in PH pathways, genes were selected in which at least 15% of the SAPH patients and no more than 5% of patients without PH carried a rare variant. Results: In the diagnostic PH gene panel, 20 different rare variants, of which 18 cause an amino-acid substitution, were detected in 23 patients: 14 SAPH patients carried a variant, as compared to 5 SA patients without PH (*p* = 0.018). Most variants were of yet unknown significance. The exploratory approach yielded five genes of interest. First, the *NOTCH3* gene that was previously linked to PH, and furthermore *PDE6B*, *GUCY2F*, *COL5A1,* and *MMP21*. Conclusions: The increased frequency of variants in PH genes in SAPH suggests a mechanism whereby the presence of such a genetic variant in a patient may increase risk for the development of PH in the context of pulmonary sarcoidosis. Replication and studies into the functionality of the variants are required for further understanding the pathogenesis of SAPH.

## 1. Introduction

Sarcoidosis is a rare granulomatous multi-organ disease of unknown cause, most commonly affecting the lungs [1]. A well-known severe complication of sarcoidosis is pulmonary hypertension (PH), defined as a mean pulmonary artery pressure of ≥25 mm Hg (mPAP ≥ 25 mm Hg) at rest as assessed by right heart catheterization [2]. Reported prevalence rates of PH in sarcoidosis patients vary widely; ranging from 3% in a prospective cohort of predominantly white patients [3] to 74% in patients awaiting lung transplantation [4]. The presence of PH in sarcoidosis is associated with an increased supplemental oxygen requirement and mortality [4,5]. Treatment regimens may include immunosuppressive or PH-targeted therapy, and should be reviewed on a case-by-case basis. According to European guidelines, PH can be clinically categorized into five groups with a similar clinical presentation, pathological findings, and hemodynamics. Sarcoidosis-associated PH (SAPH) is categorized as Group 5, PH with an unclear and/or multifactorial mechanism [2]. The underlying pathophysiological mechanism and subsequent clinical phenotype of PH in sarcoidosis are diverse [6]. A common cause of SAPH seems to be the presence of fibrosis leading to the destruction of the pulmonary vasculature. Indeed, SAPH is more common, but not exclusively present, in patients with higher Scadding stages or extensive fibrosis [3,7,8]. Other suggested mechanisms are, for example, granulomatous vasculopathy, extrinsic mechanical compression of the pulmonary vessels due to hilar lymphadenopathy or fibrosing mediastinitis, pulmonary veno-occlusive disease, portal hypertension, increased vasoreactivity, and cardiac involvement [9]. Hence, the etiology of PH in sarcoidosis is multifactorial and remains incompletely understood. Several genetic risk factors for the development of PH are known, especially for pulmonary arterial hypertension (PAH). Mutations in disease genes were found in 19.5% of the cases with sporadic PAH [10] and 54–84% of familial PAH [10,11]. The most common genetic cause of PAH is mutations in the *BMPR2* gene, encoding a receptor involved in the TGF-β superfamily [10,11,12]. However, the literature as to whether genetic variants also play a role in the development of SAPH is scarce. One case study describing four patients with severe SAPH without major fibrotic lesions demonstrated that genetic mutations may indeed be involved in the development of SAPH [13]. Research on a larger cohort would provide more insight in the role of genetic risk factors in SAPH. In the current study, we therefore describe a diverse cohort of patients with different grades of pulmonary sarcoidosis. Using a matched case-control set-up, we investigated whether rare variants in known PH risk genes are overrepresented in SAPH as compared to sarcoidosis patients without PH. To this end, we performed whole exome sequencing (WES) to identify rare variants in genes included in a diagnostic gene panel for pulmonary hypertension [14]. In addition, we used an exploratory case-control set-up to search, within 572 genes biologically involved in PH pathways, for other genes that may be involved in the development of SAPH. 

## 2. Materials and Methods

### 2.1. Patients and Samples

This study included 80 patients with pulmonary sarcoidosis from the Netherlands (*n* = 71) and the United States (*n* = 9). Diagnosis of sarcoidosis was based on the current ATS statement [15]. Half of the subjects were additionally diagnosed with PH (hereafter called the SAPH group), whereas the other 40 sarcoidosis patients did not have pulmonary hypertension (SA group). SAPH and SA patients were individually matched on sex, age, and ancestry. Pulmonary hypertension was established by right heart catheterization (RHC) with a mean pulmonary artery pressure of ≥25 mmHg, according to the 2015 European Society of Cardiology and European Respiratory Society guidelines. Absence of PH in the SA group was established based on RHC (*n* = 1) or echocardiographic assessment (*n* = 39). Subjects from the SA group underwent an echocardiogram for various reasons, including PH or cardiac sarcoidosis screening, LTX screening, or as part of a study protocol. One matched sarcoidosis patient was diagnosed with PH at follow-up after 1 year. This subject and the matched subject from the SAPH group were excluded from further analysis. The Scadding stage on the chest radiograph [16] and the extent of disease on the HRCT were recorded. 

The study was approved by the Medical research Ethics Committees United (MEC-U) of the St. Antonius Hospital (R05–08A) and the University of Cincinnati (IRB 2013–3320). All subjects signed informed consent.

### 2.2. Whole Exome Sequencing and Variant Filtering

Genomic DNA was extracted from peripheral blood using standard methods. Whole Exome Sequencing (WES) was performed by Novogene (Hong Kong, China) using the Agilent Sure-Select Human All Exon V6 kit (Agilent Technologies, Santa Clara, CA, USA) on an Illumina PE150 sequencing platform (Illumina, San Diego, CA, USA) according to standard protocol. On average, at least a 20-fold read coverage was achieved for 92.8%. Reads were aligned to reference genome assembly hg19/GRCh37, and variant call files were then uploaded into Qiagen Clinical Insight (QCI; Qiagen, Venlo, The Netherlands) software for filtering. Variants observed with a minor allele frequency (MAF) > 0.01 in gnomAD were excluded. Only variants no more than 20 bases into an intron were kept. Variants were filtered on predicted deleteriousness whereby variants were included if: (1) they are listed in HGMD; (2) if they are pathogenic, likely pathogenic, or of uncertain significance according to computed ACMG classification guidelines; or (3) if they are associated with loss of function of a gene by causing a frameshift, in-frame indel, or start/stop codon change, a missense variant, or lead to splice site loss up to 2 bases into the intron as predicted by MaxEntScan. Furthermore, only variants with a Combined Annotation-Dependent Depletion (CADD v1.6) [17] score ≥15 were included (see Table S1 for details). The remaining variants were visually inspected using the Integrative Genomics Viewer (IGV) tool to exclude variants resulting from probable sequencing artifacts.

### 2.3. Diagnostic PH Gene Panel

The diagnostic gene panel for Pulmonary Arterial Hypertension of Genomics England PanelApp version 2.16 [14] was used to select genes with a sufficient diagnostic level for a gene-disease association with PAH: *ABCC8*, *ACVRL1*, *ATP13A3*, *BMPR2*, *EIF2AK4*, *ENG*, *GDF2*, *KCNK3*, *KDR*, *SMAD9*, *SOX17*, *TBX4* (see Table S1). Additionally, *KCNA5* was added to the gene panel because a potentially pathogenic mutation in this gene was previously reported in a patient with sarcoidosis and PH [13]. 

### 2.4. Exploratory Analysis in Genes Biologically Involved in PH Pathways

Primary variant filtering steps were identical as described above. To identify genes that may have contributed to the development of PH in sarcoidosis, we subsequently used the Biological Context tool in the QCI software to select variants in 572 genes that are biologically implicated in pulmonary hypertension disease (see Table S1 for list of genes). This tool filters variants in genes known to be involved in PH based on disease models from the QIAGEN Knowledge Base, which was built with a manually curated ontology. Only genes in which at least 15% (*n* ≥ 6) of the SAPH patients and no more than 5% (*n* ≤ 2) SA patients carry a rare variant were considered for further evaluation. Vice versa, as a control, we also selected genes with *n* ≥ 6 and *n* ≤ 2 rare variant carriers in SA and SAPH patients, respectively. 

### 2.5. Statistical Analysis

Statistical analyses were performed using IBM SPSS Statistics (version 26) and R Studio (version 3.6.3). Differences between groups for continuous data were analyzed by an Independent Samples T-test or the non-parametric Mann–Whitney U test where appropriate. Categorical data were analyzed with the Pearson Chi-square or Fisher’s Exact test. Values of *p* < 0.05 were considered as statistically significant. 

## 3. Results

### 3.1. Patient Cohorts

Patients in the SAPH group had a mean mPAP on RHC of 37.4 ± 10.9 mmHg. As a result of individual matching, the two groups did not differ regarding sex, ancestry, and mean age. Significant differences, signifying more severe and fibrotic disease in SAPH, were present in the radiological Scadding stage, lung fibrosis score, and lung function parameters (Table 1).

### 3.2. Diagnostic Gene Panel

**Figure 1:**
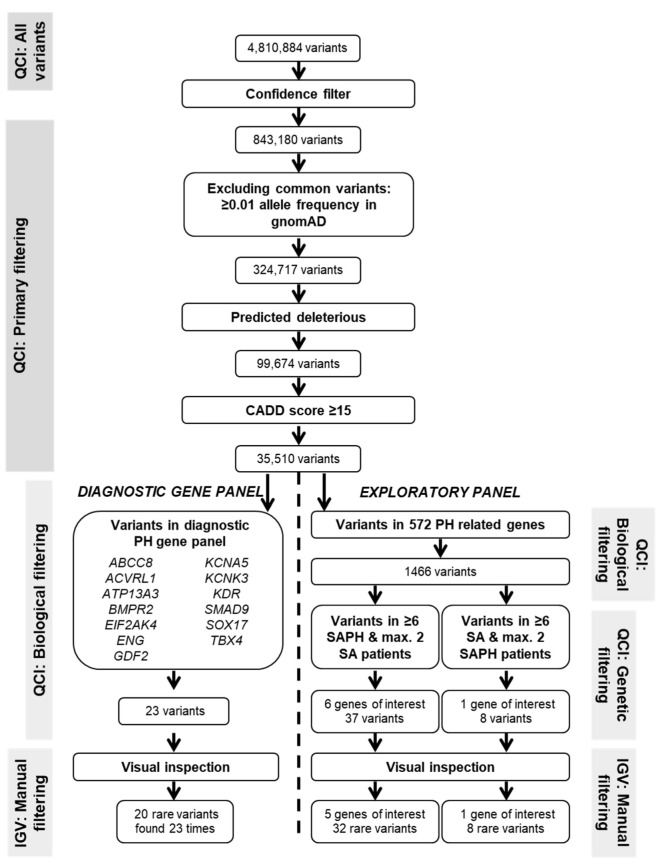
Filtering of the variants. The number of variants in the analysis after each filtering step. The first filtering steps were performed using the Qiagen Clinical Insight (QCI) software. The final step consisted of visual inspection of each variant using the Integrative Genomics Viewer (IGV) tool.

Figure 1 shows the number of variants remaining after each filtering step of the genetic analysis. Finally, twenty different rare variants were identified in the diagnostic PH gene panel and KCNA5 (see Table 2). Eighteen variants caused an amino acid substitution, and two variants (ACVRL1 c.-5–154 C > T and KDR c.3762 + 8 C > A) were intronic. Two variants were shared between patients; variant KCNA5 *p*.(R578K) was found in three different SAPH patients, and variant ACVRL1 c.-5–154C > T was present in two SAPH patients. Furthermore, two SAPH and two SA patients were each found to carry two variants. Clinical classification computed by the QCI software according to ACMG guidelines resulted in SAPH in seven variants of uncertain significance (so-called variant of unknown significance (VUS)) and six (likely) benign variants, and in SA six VUS and one likely benign variant. No (likely) pathogenic variants were found. 

In total, 35.9% (14/39) of the SAPH patients carried at least one rare variant in a gene from the diagnostic panel, as compared to 12.8% (5/39) of the SA patients (*p* = 0.018). Baseline characteristics regarding lung abnormalities or hemodynamics did not differ between patients with and without a variant in the SAPH and in the SA group, though rare variants were more frequently present in white patients than in non-white patients (48.1% vs 8.3%; *p* = 0.03) (see Supplemental Table S1). There was no difference in the frequency of carrying a variant between SAPH patients without extensive pulmonary fibrosis on chest imaging (Scadding stage 0–3) and patients with fibrosis (Scadding stage 4) (*p* = 0.73).

### 3.3. Genes of Biological Relevance in PH

To explore the presence of rare variants in genes with biological relevance to PH pathogenesis, we analyzed 572 genes. The selection of genes that have rare variants in at least six SAPH patients and a maximum of two SA patients yielded five genes of interest: *NOTCH3* (11 variants), *PDE6B* (9 variants), the X-chromosomal *GUCY2F* (5 variants), *COL5A1* (4 variants), and *MMP21* (3 variants). A total of 32 variants was found in these genes, all missense variants resulting in amino acid substitutions (see Table 3). A total of 21 SAPH patients and 6 SA patients carried at least one variant in these genes. Nine SAPH patients and one SA patient carried a variant in more than one gene, mostly two genes. 

Conversely, selecting biologically relevant PH genes in which at least six SA patients and not more than two SAPH patients carry a variant yielded one gene, *PDE4A* (Phosphodiesterase 4A; see Supplemental Table S3). Six SA patients and two SAPH patients carried one variant in this gene.

## 4. Discussion

To our knowledge, we here present the largest genetic study of sarcoidosis-associated pulmonary hypertension so far. In a diagnostic PAH panel of 13 genes, we found a significantly higher number of rare variants in SAPH patients than in sarcoidosis patients without PH. In addition, in an exploratory approach, we found five genes in which 15% or more patients with SAPH carried a rare variant within 572 genes involved in PH pathogenesis: *NOTCH3*, *PDE6B*, *GUCY2F*, *COL5A1*, and *MMP21.*

The case-control set-up demonstrated that rare variants in the diagnostic panel were significantly more frequent in sarcoidosis patients with PH as compared to those without PH. Interestingly, the variants identified in the current study were not classified as pathogenic according to the ACMG guidelines [22]. Of note, the ACMG classification is used in clinical genetics for clinical decision-making: only in cases of a high degree of certainty about the pathogenicity of the variant are clinical consequences allowed. Therefore, only variants with high penetrance are considered (likely) pathogenic [22]. Although the variants identified here do not meet these criteria, all passed our filtering steps, including having a CADD score of at least 15, indicative of a probable deleterious effect. In congruence with this, most observed variants are predicted to cause amino acid substitutions. It is hence well possible that our variants do modify protein functioning and influence pathways involved in PH. 

Several of the variants were described before. *ENG* p.(G191D) was found in 10 PAH patients by Pousada et al., whereby 9 out of 10 cases carried a second variant in *BMPR2*, *ACVRL1*, or *KCNA5* [23]. It was also found in two Dutch families with hereditary hemorrhagic telangiectasia (HHT), but the role of this variant in the development of disease was unclear [24]. In the current study, we found this variant in one SAPH patient who additionally carried a variant in *KCNA5* (p.(R578K)). This KCNA5 variant was previously described in patients with atrial fibrillation, but also in some healthy controls [25,26]. Functionally, the p.R578K variant has a significant but minor effect on channel gating and results in resistance to drug blocking [27]. Another variant that was found in *ENG*, p.(S615L) was observed in patients with HHT in numerous studies, often in combination with another variant in *ENG* or *ACVRL1* [28,29,30,31]. Functional cell experiments on the p.S615L variant however showed no change in subcellular localization and no defect in the BMP9/ALK1 signaling pathway [28,32]. The *ACVRL1* p.(A482V) variant was previously observed in multiple HHT studies, but, as supported by in vitro results, it is suggested to be a rare benign polymorphism [33,34,35,36].

Among the *KDR* variants that we detected, p.(V136M) was previously reported heterozygous in an Italian patient with vascular anomalies as a variant of uncertain significance [37]. The *SMAD9* variant identified here in a patient without PH, p.(R226Q), was previously found in a patient with PAH who also carried a *BMPR2* variant [38]. *TBX4* p.(A35V) was previously found in a Dutch patient with adult-onset PAH and was classified as a variant of uncertain significance (VUS) [39]. However, this variant was here observed in a SA patient without PH. It therefore remains to be determined whether this patient is at increased risk of developing PH. 

Interestingly, only one SAPH patient (2.6%) was found to carry a variant in *BMPR2*, whereas mutations in *BMPR2* are the most common genetic cause of disease in both heritable (~75% of patients) [11,12] and in those initially diagnosed as idiopathic (12–14% of the patients) PAH [10,11]. Disease penetrance of pathogenic *BMPR2* variants in hereditary PAH is approximately 27%, with female penetrance being three-fold that of male penetrance (42% and 14%, respectively [40]). Mutations in other PAH-related genes are rare with reported frequencies that are usually well below 1% [11,12].

A previous case study with four SAPH patients found that two of the four patients carried a mutation in *BMPR2*. However, these patients had a disease phenotype distinct from the majority of SAPH patients in our study, with severe PH (mPAP > 50 mmHg) and mild pulmonary involvement [13]. Our low frequency of *BMPR2* variants is more in line with small studies on PH associated with connective tissue disease (CTD). Genotyping for 17 mutations in *BMPR2* that cause primary PH did not detect any mutation in 12 patients with CTD [41], whereas Morse et al. found one potentially benign *BMPR2* variant in a study of 24 patients with PH and a scleroderma spectrum of disease [42]. 

Our study indicates that currently recognized pathogenic mutations in the known PAH genes are not a common cause of SAPH. The rare variants of yet unknown significance that we identified in the diagnostic panel of genes might increase risk for the development of PH only in the context of sarcoidosis. Multiple variants observed here were previously reported, though usually without compelling evidence for pathogenicity and sometimes in combination with a second variant. This suggests that the variants in PH-associated genes that we detected in our SAPH cohort are not deleterious in healthy subjects but act as risk factors for the development of PH in the context of pulmonary sarcoidosis.

Searching for biologically relevant genes with increased variant frequency in SAPH patients yielded five genes of interest. Further research including replication is required before any causal role of these genes in SAPH can be inferred. Strikingly, the gene with the most rare variants in the SAPH group, *NOTCH3*, has been directly linked to PH before [43]. Two variants in the *NOTCH3* gene were reported in childhood-onset PAH. Mutant cell lines showed that these two variants impaired NOTCH3 activity upon stimulation with its ligand Jagged1 and increased cell proliferation and viability. NOTCH3 protein levels were reduced, probably due to increased clearance speed [43]. NOTCH3 is highly expressed in vascular smooth muscle cells where it promotes cell proliferation, differentiation, and survival [44].The variant that was found in the two SA patients was actually predicted to have a gain-of-function effect, whereas the predicted effect of the variants in the SAPH patients was normal function or loss of function. However, increased *NOTCH3* expression was observed in lung tissue in PAH patients, and increased levels correlated with pulmonary vascular resistance as a measure of disease severity [45]. Furthermore, Li et al. (2009) showed that *NOTCH3* knockout mice, in contrast to wildtypes and heterozygotes, did not develop PH in response to hypoxic stimulation [45]. These findings point to a possible role for *NOTCH3* in PH disease, and functional studies of the variants in this study are therefore of considerable interest.

Our study has several limitations. PH is a dynamic pathophysiological disorder, and it cannot be excluded that patients without PH at the time of the study may develop this at a later stage, exemplified by the SA patient without PH who was excluded from the study because of development of PH at a later time. Time between diagnosis of sarcoidosis and development of PH was found to be 12–17 years on average [3,46,47], whereas Baloira Villar et al. noted that this period was 4–6 years in their four-patient case study [13]. Furthermore, patients were not matched for disease severity or duration. We found PH displayed in patients with higher Scadding stages and more pulmonary fibrosis compared to patients without PH in our cohort. This is in line with previous studies [3,7,8] and represents the population as that which would be encountered in the clinic. Additionally, large deletions may have been missed in this study due to the method that was used. With the current small sample size and design, the findings are hypothesis generating. Hence, replication of these findings in other cohorts of SAPH patients are needed and would be of utmost interest. 

Knowledge regarding the involvement of genetics in the development of SAPH is limited. The current study shows that genetic variants in genes implicated in PH are also present in SAPH patients with a variety of clinical phenotypes and are significantly more frequent than in sarcoidosis patients without PH. Therefore, we hypothesize that variants in PAH-associated genes may also play a role in the development of sarcoidosis-associated PH. Interestingly, several variants were also previously reported in PH patients, though not deemed pathogenic. Our findings hence suggest that SAPH patients do not carry variants that cause hereditary PAH but do carry rare variants in PH genes that may predispose them to the development of PH in the presence of existing pulmonary inflammation or damage. However, before translation to the clinic, further research to confirm the excess of rare variants in PH genes in SAPH patients as well as to determine their functional consequence is needed. 

## Figures and Tables

**Table 1:** Baseline characteristics of SAPH and SA patients. FVC % predicted: forced vital capacity % of predicted value; DLCO SB % predicted: single-breath diffusing capacity of the lung for carbon monoxide % predicted; Mean PAP: mean pulmonary arterial pressure; PVR: pulmonary vascular resistance; PCWP: pulmonary capillary wedge pressure; ND: parameter was not determined.

		SAPH (*n* = 39)	SA (*n* = 39)	*p*-Value
Male, *n* (%)		19 (48.7)	19 (48.7)	1.00
Age mean (SD), y		59.2 (11.3)	60.9 (10.9)	0.50
Ancestry	White, *n* (%)	27 (69.2)	27 (69.2)	1.00
	Black, *n* (%)	9 (23.1)	9 (23.1)	
	Hindustan, *n* (%)	3 (7.7)	3 (7.7)	
Scadding stage (*n* = 38; *n* = 39)	0, *n* (%)	2 (5.3)	8 (20.5)	0.016
	1, *n* (%)	4 (10.5)	3 (7.7)	
	2, *n* (%)	4 (10.5)	9 (23.1)	
	3, *n* (%)	2 (5.3)	6 (15.4)	
	4, *n* (%)	26 (68.4)	13 (33.3)	
Lung fibrosis score (*n* = 38; *n* = 39)	<5%, *n* (%)	9 (23.7)	22 (56.4)	<0.001
	5–20%, *n* (%)	1 (2.6)	7 (17.9)	
	>20%, *n* (%)	28 (73.7)	10 (25.6)	
FVC % predicted, mean (SD) (*n* = 37; *n* = 36)		66.6 (19.4)	94.2 (23.1)	<0.001
DLCO SB% predicted, mean, mean (SD) (*n* = 32; *n* = 32)		47.8 (18.5)	68.4 (15.2)	<0.001
Mean PAP mmHg, mean (SD)		37.4 (10.9)	ND	
PVR Wood units, mean (SD) (*n* = 36; ND)		5.6 (3.1)	ND	
Cardiac output L·min-1, mean (SD) (*n* = 38; ND)		5.7 (1.8)	ND	
PCWP mmHg, mean (SD) (*n* = 37; ND)		9.7 (3.6)	ND	

**Table 2:** Variants in the diagnostic gene panel (a) SIFT: T= Tolerated; D = Damaging; A = Activating; (b) PolyPhen-2: B= Benign; PrD = Probably Damaging; PoD = Possibly Damaging; (c) Mutation Taster: D = Deleterious; B = Benign; (d) Mutation Assessor functional impact: N= Neutral; L= Low; M= Medium; H= High; Predicted functional impact high or medium is predicted functional; low or neutral is predicted non-functional. Predictions by SIFT [18], PolyPhen-2 [19], Mutation Taster2021 [20], or Mutation Assessor [21] indicating deleterious consequences are underlined. Carriers are of European ancestry except SAPH6 and SA41 (Hindustan) and SA71 (African American). (e) Variant classification according to ACMG 2015 guidelines as provided by QCI; P = Pathogenic; LP = Likely Pathogenic; VUS = Variant of Uncertain Significance; LB = Likely Benign; B = Benign. NA = not applicable.

Gene	Transcript	Nucleotide Change	Amino Acid Change	dbSNP	gnomAD Frequency	CADD	SIFT ^a^	PPh2 ^b^	Mutation Taster ^c^	Mutation Assessor ^d^	Variant Classification ^e^	SAPH Carrier ID(s)	SA Carrier ID(s)
*KCNK3*	NM_002246.3	c.418C>A	p.(L140M)	rs755486166	0.0000	25.5	D	PrD	D	M	VUS	-	SA67
*BMPR2*	NM_001204.7	c.86A>G	p.(N29S)	rs112862820	0.0007	16.2	T	B	B	N	LB	SAPH5	-
*KDR*	NM_002253.4	c.3762+8C>A	NA	rs78801899	0.0003	17.0	NA	NA	B	NA	VUS	-	SA71
*KDR*	NM_002253.4	c.2854G>A	p.(V952I)	rs13129474	0.0000	20.0	T	B	B	N	VUS	-	SA41
*KDR*	NM_002253.4	c.2837G>A	p.(R946H)	rs140041720	0.0002	28.7	D	PrD	B	N	VUS	SAPH1	-
*KDR*	NM_002253.4	c.406G>A	p.(V136M)	rs35636987	0.0007	24.1	D	PrD	B	M	VUS	SAPH6	-
*KDR*	NM_002253.4	c.170G>C	p.(R57T)	rs139047809	0.0012	19.3	T	PoD	B	M	VUS	-	SA41
*ENG*	NM_000118.3	c.1844C>T	p.(S615L)	rs148002300	0.0015	21.1	T	B	B	M	LB	SAPH10	-
*ENG*	NM_000118.3	c.583G>A	p.(E195K)	rs1255912441	0.0000	17.6	T	B	B	M	VUS	SAPH15	-
*ENG*	NM_000118.3	c.572G>A	p.(G191D)	rs41322046	0.0091	24.2	D	PrD	B	M	B	SAPH11	-
*KCNA5*	NM_002234.4	c.188G>T	p.(G63V)	rs768062067	0.0000	20.1	-	B	B	N	VUS	SAPH34	-
*KCNA5*	NM_002234.4	c.929C>T	p.(P310L)	rs17215402	0.0040	18.2	T	B	B	L	LB	SAPH12	-
*KCNA5*	NM_002234.4	c.1733G>A	p.(R578K)	rs12720445	0.0054	17.2	T	B	B	M	LB	SAPH24; SAPH13; SAPH11	-
*ACVRL1*	NM_001077401.2	c.-159C>T	NA	rs542225698	0.0009	17.3	NA	NA	B	NA	VUS	SAPH23; SAPH8	-
*ACVRL1*	NM_000020.3	c.1445C>T	p.(A482V)	rs139142865	0.0016	23.2	D	PrD	B	M	LB	SAPH26	-
*SMAD9*	NM_005905.6	c.677G>A	p.(R226Q)	rs78249575	0.0012	17.9	A	B	B	N	VUS	-	SA77
*EIF2AK4*	NM_001013703.4	c.13C>T	p.(R5C)	rs746773845	0.0000	23.3	NA	B	B	L	VUS	SAPH10	-
*EIF2AK4*	NM_001013703.4	c.4792G>A	p.(A1598T)	rs1045326564		21.0	T	B	B	N	VUS	-	SA71
*TBX4*	NM_018488.3	c.104C>T	p.(A35V)	rs148424252	0.0089	16.5	T	B	B	N	LB	-	SA58
*TBX4*	NM_018488.3	c.1094A>G	p.(H365R)	rs1443274012	0.0000	25.5	T	PrD	D	M	VUS	SAPH17	-

**Table 3:** Rare variants found in the exploratory case-control analysis in 572 genes biologically involved in PH pathogenesis. Genes biologically involved in PH pathways in which ≥15% of the SAPH patients carry a variant and no more than 5% (*n* = 2) of the SA patients do. (a) SIFT: T = Tolerated; D = Damaging; A = Activating; (b) PolyPhen-2: B= Benign; PrD = Probably Damaging; PoD = Possibly Damaging; (c) Mutation Taster: D = Deleterious; B = Benign; (d) Mutation Assessor functional impact: N = Neutral; L = Low; M = Medium; H = High; Predicted functional impact high or medium is predicted functional; low or neutral is predicted non-functional. Predictions by SIFT, PolyPhen-2, Mutation Taster, or Mutation Assessor indicating deleterious consequences are underlined. (e) Variant classification according to ACMG 2015 guidelines; P = Pathogenic; LP = Likely Pathogenic; VUS = Variant of Uncertain Significance; LB = Likely Benign; B = Benign. * D in transcript ENST00000429163.

Gene	Transcript	Nucleotide Change	Amino Acid Change	dbSNP	gnomAD Frequency	CADD	SIFT ^a^	PPh2 ^b^	Mutation Taster ^c^	Mutation Assessor ^d^	Variant Classification ^e^	SAPH Carrier ID(s)	SA Carrier ID(s)
*PDE6B*	NM_001145291.2	c.35T>C	p.(L12P)	-	-	24.1	-	PrD	D	M	VUS	SAPH20	-
*PDE6B*	NM_001145291.2	c.143G>A	p.(R48Q)	rs113842820	0.0082	15.4	-	B	B	L	B	SAPH1; SAPH38	-
*PDE6B*	NM_001145291.2	c.373C>G	p.(P125A)	rs28414606	0.0078	15.6	-	B	B	N	B	SAPH38	-
*PDE6B*	NM_001145291.2	c.496G>A	p.(E166K)	rs115775983	0.0090	15.6	-	B	B	N	LB	SAPH1	SA68
*PDE6B*	NM_001145291.2	c.706G>A	p.(G236S)	rs75543439	0.0002	20.4	-	B	B	N	VUS	SAPH14	-
*PDE6B*	NM_001145291.2	c.794G>A	p.(R265Q)	rs144562730	0.0011	26.1	-	PrD	B	L	VUS	SAPH12	-
*PDE6B*	NM_001145291.2	c.1295C>T	p.(T432I)	rs775120495	0.0000	23.7	-	PoD	B *	M	VUS	SAPH34	-
*PDE6B*	NM_001145291.2	c.2344G>A	p.(V782M)	rs145124626	0.0003	26.9	-	PrD	D	M	VUS	-	SA41
*PDE6B*	NM_001145291.2	c.2524G>A	p.(G842S)	rs367709559	0.0000	22.3	-	B	B	L	VUS	-	SA41
*COL5A1*	NM_001278074.1	c.278C>T	p.(A93V)	rs41306397	0.0087	22.2	D	B	B	L	LB	SAPH28; SAPH16	SA61
*COL5A1*	NM_001278074.1	c.2695G>A	p.(G899S)	rs149964491	0.0016	23.3	T	PoD	B	N	LB	SAPH38	-
*COL5A1*	NM_001278074.1	c.4906G>A	p.(A1636T)	rs113452150	0.0073	29.1	D	PoD	D	H	VUS	SAPH40	-
*COL5A1*	NM_001278074.1	c.5407G>A	p.(D1803N)	rs61729495	0.0061	19.0	A	B	B	N	LB	SAPH30; SAPH5	-
*MMP21*	NM_147191.1	c.1361C>T	p.(A454V)	rs28381319	0.0002	22.3	T	B	B	M	B	SAPH31; SAPH35; SAPH39	SA80
*MMP21*	NM_147191.1	c.1046A>G	p.(E349G)	rs28381302	0.0021	23.2	D	PoD	B	M	LB	SAPH21; SAPH12	SA54
*MMP21*	NM_147191.1	c.290C>T	p.(A97V)	rs554501102	0.0006	17.4	D	B	B	M	VUS	SAPH27	-
*NOTCH3*	NM_000435.3	c.6221C>T	p.(P2074L)	rs114447350	0.0003	21.9	T	B	B	N	B	SAPH33; SAPH30	-
*NOTCH3*	NM_000435.3	c.5900T>C	p.(M1967T)	rs377589088	0.0012	26.9	D	PrD	D	N	VUS	SAPH28	-
*NOTCH3*	NM_000435.3	c.5854G>A	p.(V1952M)	rs115582213	0.0088	27.3	D	PrD	B	L	B	-	SA48; SA68
*NOTCH3*	NM_000435.3	c.5284G>A	p.(V1762M)	rs756495084	0.0030	15.5	T	B	B	N	LB	SAPH27	-
*NOTCH3*	NM_000435.3	c.4762A>C	p.(N1588H)	-	0.0009	26.3	D	PrD	B	M	VUS	SAPH11	-
*NOTCH3*	NM_000435.3	c.4679G>C	p.(R1560P)	rs78501403	0.0097	24.7	T	PrD	B	L	LB	SAPH35	-
*NOTCH3*	NM_000435.3	c.3352A>T	p.(N1118Y)	rs376950447	0.0001	22.3	D	PrD	B	L	VUS	SAPH24	-
*NOTCH3*	NM_000435.3	c.3088G>A	p.(G1030R)	rs1179899018	0.0082	23.8	D	PrD	D	H	VUS	SAPH21	-
*NOTCH3*	NM_000435.3	c.2039G>A	p.(R680H)	rs10406745	0.0002	17.3	T	B	B	L	LB	SAPH30	-
*NOTCH3*	NM_000435.3	c.1490C>T	p.(S497L)	rs114207045	-	24.9	T	B	B	L	B	SAPH9	-
*NOTCH3*	NM_000435.3	c.182G>A	p.(R61Q)	rs1222763947	0.0015	18.0	T	B	B	N	VUS	SAPH12	-
*GUCY2F*	NM_001522.3	c.2380G>A	p.(E794K)	rs35726803	0.0001	28.5	D	PrD	B	M	VUS	SAPH24	SA68
*GUCY2F*	NM_001522.3	c.1883G>A	p.(R628Q)	rs7883913	0.0000	24.1	D	PrD	B	M	VUS	SAPH12	-
*GUCY2F*	NM_001522.3	c.1720A>C	p.(K574Q)	rs139586665	0.0048	25.2	D	PrD	B	M	VUS	SAPH29	-
*GUCY2F*	NM_001522.3	c.1483A>C	p.(N495H)	rs148768857	0.0067	20.2	T	B	B	L	LB	SAPH40; SAPH39	-
*GUCY2F*	NM_001522.3	c.688C>T	p.(R230W)	rs33973457	0.0000	22.7	D	PoD	B	L	LB	SAPH33	-

## Data Availability

Data can be requested from the corresponding author.

## Supplementary Materials

The following supporting information can be downloaded at: https://www.mdpi.com/article/10.3390/diagnostics12102564/s1, Table S1: Baseline characteristics of SAPH patients with a variant in diagnostic gene panel compared to SAPH patients without a variant; Table S2: Baseline characteristics of SA patients with a variant in diagnostic gene panel compared to SA patients without a variant. Table S3: Overview of rare variants in biologically relevant genes in which ≥15% of the SA patients carry a rare variant.
