# Supplementary material for: Pulmonary Hypertension Associated Genetic Variants in Sarcoidosis Associated Pulmonary Hypertension
Source: Diagnostics (Basel). 2022 Oct 21;12(10):2564. doi: 10.3390/diagnostics12102564 (PMC9601358; doi:10.3390/diagnostics12102564)
Supplement: Supplementary file 1 [file diagnostics-12-02564-s001.zip › diagnostics-1924163-supplementary_proof readv2.pdf]

SUPPLEMENTAL INFORMATION
Pulmonary hypertension associated genetic variants in sarcoidosis associated pulmonary hypertension

Supplemental Information S1. Methods

Variant filtering

Variant call files were uploaded into QIAGEN Clinical Insight Interpret Translational (QCI; Qiagen) software for filtering. Only variants were kept with a call quality of ≥20, read depth ≥10, genotype quality ≥30 and allele fraction ≥25 in any case or in any control. Variants observed with an allele frequency of at least 1 in 100 in gnomAD were excluded. Only variants no more than 20 bases into an intron were kept. Variants were filtered on predicted deleteriousness whereby variants were included if 1) they are listed in HGMD; or 2) if they are pathogenic, likely pathogenic or of uncertain significance according to computed ACMG classification guidelines; or 3) if they are associated with loss of function of a gene by causing a frameshift, in-frame indel, or start/stop codon change, a missense variant, or lead to splice site loss up to 2 bases into intron as predicted by MaxEntScan. Any remaining variants with a Combined Annotation-Dependent Depletion (CADD v1.6) score <15 were excluded.

Additional predictions of variant pathogenicity by the PolyPhen-2 (v2.2.2[19]), SIFT (BSIFT 2016-02-23)[18], Mutation Assessor (release 3)[21] and Mutation Taster2021[20] software are displayed for information but were not used as filtering criteria. These tools predict the effect of variants based on evolutionary conservation and the effect on protein structure and function. CADD, SIFT and PolyPhen-2 predictions are incorporated in the QCI software, whereas Mutation Assessor and MutationTaster2021 were accessed through their web-based software. CADD integrates various different annotations into one Phred-based score of deleteriousness for single nucleotide variants (SNVs) and indels[17]. Predicted variant classification according to American College of Medical Genetics and Genomics (ACMG) 2015 guidelines[22] was also extracted from QCI software.

Variants resulting from probable sequencing artefacts were excluded by means of visual inspection of the raw BAM file in IGV v2.8. Three variants were excluded from further analysis after visual examination in IGV (all deletions or insertions in a series of trinucleotide repeats in the *BMPR2* 5'UTR region)

Clinical gene panel for P(A)H

The clinical panel was composed based on the information from the Pulmonary Arterial Hypertension of Genomics England PanelApp version 2.16[14]. It consists of seven genes that were rated as diagnostic level of evidence (listed as 'Green') on the Pulmonary Arterial Hypertension of Genomics England PanelApp version 2.16 (i.e. *ACVRL1, ATP13A3, BMPR2, EIF2AK4, ENG, GDF2, KCNK3*, *SMAD9*, *SOX17*, *TBX4*). Additionally, two genes listed as 'Amber' (evidence for gene-disease association is moderate) were included because Expert reviews state there is now sufficient evidence to classify the genes as 'Green' (*ABCC8* and *KDR*)[14]. *KCNA5* did not meet above-mentioned criteria but was included because a potentially pathogenic mutation in this gene was previously identified in a patient with sarcoidosis and PH[13].

Biologically relevant genes with variants in ≥15% of SAPH patients

Initial filtering steps were the same as the clinical panel, with the exception of the clinical panel itself. Instead, several steps were taken in QCI to yield a list of genes in which at least six SAPH patients carry a variant and a maximum of two SA patients. First, the Biological Context tool in the QCI software was used to select variants in 572 genes that are implicated in pulmonary hypertension disease (see SI.1 for

list of genes). From this list of genes, a list was created of genes in which at least three SA patients carry a variant. This was done with the Genetic Analysis tool that can select genes with a (homo-, hetero-, or hemizygous, or het-ambiguous) variant in at least three SA patients on the gene level. To yield the genes of interest, after initial filtering 1) only variants in 572 PH-associated genes were selected (Biological Context tool); 2) genes with three or more variant carriers among SA patients were excluded (by uploading the previously created list in a second Biological Context tool); and 3) genes in which at least 6 SAPH patients carry a (homo-, hetero-, or hemizygous, or het-ambiguous) variant on the gene level were selected using the Genetic Analysis tool. The remaining genes are the genes of interest with variants in ≥15% of SAPH patients and maximally two of the SA patients. The complete list of variants is then obtained by 1) initial filtering; 2) selecting variants in 572 PH-associated genes (Biological Context tool); and 3) selecting the genes of interest in a separate Biological Context tool).

572 genes implicated in pulmonary hypertension according to QCI software

A4GNT	BBS4	CHD7	DHX38	FLT1	GUCY2D
ABCA3	BBS5	CHID1	DNAAF2	FLT4	GUCY2F
ABCC1	BBS7	CHRNA1	DNAH5	FOSL2	GZMB
ABCC8	BCL2L1	CHRNA10	DNMT3A	FOXC2	H1-2
ABCC9	BICC1	CHRNA2	DOCK6	FOXD3	HBB
ABL1	BIRC5	CHRNA3	DRC1	FOXF1	HBP1
ACTA2	BMP10	CHRNA4	DYNC2H1	FOXO1	HGF
ACTC1	BMP2	CHRNA5	DYNC2I1	FREM2	HIRA
ACVR2B	BMP4	CHRNA6	DYNC2I2	FRZB	HLA-DQA1
ACVRL1	BMP8A	CHRNA7	ECE1	FTSJ3	HLA-DQB1
ADA	BMPR1A	CHRNA9	EDN1	G6PC3	HLA-DRB1
ADAMTS1	BMPR1B	CHRNB1	EDNRA	GAB1	HLA-DRB5
ADAMTS18	BMPR2	CHRNB2	EDNRB	GABRA1	HRG
ADAMTS6	BRAP	CHRNB3	EFTUD2	GABRA2	HTR2A
ADORA2A	BRME1	CHRNB4	EGFR	GABRA3	HTR2B
ADORA2B	BROX	CHRND	EGLN1	GABRA4	IFNA1/IFNA13
ADRA2A	BRWD3	CHRNE	EID1	GABRA5	IFNA10
ADRA2B	C3orf18	CHRNG	EIF2AK4	GABRA6	IFNA14
ADRA2C	C7orf31	CHST9	ELL	GABRB1	IFNA16
ADRB1	C9	CHUK	ELN	GABRB2	IFNA17
ADRB2	CA1	CLCN5	EMC8	GABRB3	IFNA2
AFF2	CA10	CLMP	EMID1	GABRD	IFNA21
AGT	CA11	CMYA5	ENG	GABRE	IFNA4
AGTR1	CA12	CNDP1	ENPP6	GABRG1	IFNA5
AGTR2	CA13	CNTLN	EOGT	GABRG2	IFNA6
AHR	CA14	CNTN4	EP300	GABRG3	IFNA7
ALDH9A1	CA2	CNTRL	EPAS1	GABRP	IFNA8
ALG3	CA3	COL18A1	EPC1	GAMT	IFNAR1
ALPG	CA4	COL1A2	EPHB4	GDE1	IFNB1
ALS2	CA5A	COL27A1	ERG	GDF2	IFNE
AMOT	CA5B	COL2A1	ESRRA	GDPD1	IFNG
ANGPT1	CA6	COL3A1	EXOC6B	GDPD3	IFNK
ANK3	CA7	COL5A1	EZH1	GDPD4	IFNW1
APEX1	CA8	COL5A2	F2	GGCX	IFT140
APOE	CA9	CPLANE1	F5	GIPC2	IL12B
AQP1	CACNA1C	CPS1	F8	GLRA1	IL18
AR	CARNS1	CRACR2A	FA2H	GLRA2	IL1R1
ARG1	CAV1	CREBBP	FAM149A	GLRA3	IL1RN
ARG2	CBLN2	CSF1R	FARSA	GLRA4	IMMT
ASH2L	CCDC39	CSF2	FBLN2	GLRB	INHBA
ASS1	CCDC40	CSF2RA	FBN1	GPR174	INHBB
ATF6	CCDC50	CSNK2A2	FBN2	GPSM2	INHBC
ATG10	CCL2	CX3CL1	FCN2	GRHL2	INHBE
ATP13A3	CCR7	CXCR4	FGA	GRIA1	INVS
ATP2C1	CCRL2	DAB2	FGB	GRM8	ITGAM
ATP6V0A2	CD22	DBF4B	FGF12	GUCY1A1	ITPR1
BAZ1B	CD248	DCAF12	FHL1	GUCY1A2	ITPR3
BBS10	CFTR	DDR1	FICD	GUCY1B1	ITSN1
BBS2	CHD4	DDR2	FLNA	GUCY2C	JAG1

KCNA5
KCNH2
KCNK3
KDM3B
KDR
KEAP1
KIAA2026
KIF21A
KIF24
KIF7
KIT
KLB
KLF2
KLK1
KMT2D
L1CAM
LAMA1
LAMA4
LAMC1
LIFR
LIN7A
LIPH
LLGL1
LOXL3
LRP1
LRP2
LTBP1
LZTR1
MAP2K1
MAP4
MAPK6
MAT2A
MBTPS1
MCAM
MCM3AP
MEFV
MEIS1
MESD
MIF
MIR204
MIR21
MIR424
MIR503
MKKS
MMACHC
MME
MMP21
MN1
MPPE1
MPPED2
MRTFA
MSH2
MST1
MTHFR
MUC5B
MYH2
MYH6
MYO16
MYO5C
NAPEPLD
NCK1
NDST1
NEK1
NEK8
NEU1
NF1
NFKBIA
NIPBL
NKX2-5
NKX2-6
NOB1
NOS2
NOS3
NOTCH1
NOTCH2
NOTCH3
NOX4
NPPA
NPPB
NPR1
NPR2
NPR3
NR3C2
NSD1
NSDHL
NSMAF
NUCB1
NUDCD1
NVL
OPA3
OPLAH
OR2T34
OSGEPL1
OTC
P2RY12
PCARE
PCOLCE2
PDCD4
PDE10A
PDE11A
PDE12
PDE1A
PDE1B
PDE1C
PDE2A
PDE3A
PDE3B
PDE4A
PDE4B
PDE4C
PDE4D
PDE5A
PDE6A
PDE6B
PDE6C
PDE6D
PDE6G
PDE6H
PDE7A
PDE7B
PDE8A
PDE8B
PDE9A
PDGFD
PDGFRA
PDGFRB
PEX1
PEX13
PFKFB3
PGS1
PHF12
PHF20L1
PIM1
PITX2
PLA2G2D
PLAG1
PLAT
PLD6
PLG
PLOD1
POLG
POU5F1
PPARD
PPIA
PPL
PPP1R13L
PPP2R3A
PRDM1
PRDM9
PREX1
PRICKLE1
PRKG1
PRMT2
PSMC4
PTGDR2
PTGER1
PTGIR
PTGIS
PTPN11
PTPN14
RAB23
RAF1
RAI1
RARS2
RASA2
RBL2
RET
REV3L
RNF213
ROBO1
ROBO2
ROR2
RPTOR
RYR2
S100A1
SAMHD1
SARS2
SCAF11
SCN10A
SCN1A
SCN1B
SCN5A
SCN9A
SEC23IP
SELP
SEMA6D
SERPINE1
SH3TC2
SHH
SIRT3
SLC25A24
SLC2A1
SLC2A10
SLC2A4
SLC6A17
SLC6A4
SLC8A1
SLC9A1
SMAD1
SMAD4
SMAD6
SMAD7
SMAD9
SMARCA2
SMARCA4
SMARCAL1
SMPDL3A
SMPDL3B
SMS
SMYD4
SOD1
SOX17
SPATC1
SPTA1
SRC
SS18
TAF13
TBC1D10B
TBX20
TBX4
TCN2
TDP2
TEK
TET2
TF
TGFB1
TGFBI
THBD
THBS1
THSD7A
THYN1
TM4SF20
TMEM67
TMEM70
TNC
TNF
TNFAIP3
TNFAIP6
TNFRSF13B
TNFSF10
TNFSF4
TNNC1
TNNC2
TNNI1
TNNI2
TNNI3
TNNT1
TNNT2
TNNT3
TOP1
TOPBP1
TOR1B
TRH
TRPC1
TRPC6
TSC1
TSC2
TSHZ1
TTLL3
TUBB6
TULP2
VANGL1
VCAN
VCL
VHL
VIM
VIP
WDFY4
WDR5
WNK1
WNT1
WRN
ZADH2
ZMYM2
ZNF146
ZNF280B
ZNF620
ZSWIM9

SUPPLEMENTAL DATA

Supplemental Table S1 Baseline characteristics of SAPH patients with a variant in diagnostic gene panel compared to SAPH patients without a variant

		**SAPH – no variant in diagnostic gene panel (n=25)**	**SAPH – with variant in diagnostic gene panel (n=14)**	**p-value**
**Male, n (%)**		12 (48.0)	7 (50.0)	0.90
**Age mean (SD), y** (n=25; n=13)		59.9 (10.4)	59.7 (11.9)	0.96
**Ancestry %**	White, n (%)	14 (56.0)	13 (92.9)	0.018
	Black, n (%)	9 (36.0)	0 (0.0)	
	Hindustan, n (%)	2 (8.0)	1 (7.1)	
**Scadding stage** (n=24; n=14)	0, n (%)	2 (8.3)	0 (0.0)	0.55
	1, n (%)	3 (12.5)	1 (7.1)	
	2, n (%)	1 (4.2)	3 (21.4)	
	3, n (%)	1 (4.2)	1 (7.1)	
	4, n (%)	17 (70.8)	9 (64.3)	
**Lung fibrosis score**	< 5%, n (%)	5 (20.8)	4 (28.6)	0.50
	5-20%, n (%)	0 (0.0)	1 (7.1)	
	>20%, n (%)	19 (79.2)	9 (64.3)	
**FVC % predicted, mean (SD)** (n=24; n=13)		65.3 (19.1)	69.0 (20.6)	0.22
**DLCO SB% predicted, mean, mean (SD)** (n=20; n=12)		49.0 (17.4)	45.8 (20.8)	0.64
**Mean PAP mmHg, mean (SD)**		38.3 (11.6)	36.0 (9.8)	0.59
**PVR Wood units, mean (SD)** (n=24; n=12)		6.0 (3.3)	4.8 (2.5)	0.56
**Cardiac output L·min-1, mean (SD)** (n=25; n=13)		5.8 (2.0)	5.6 (1.3)	0.88
**PCWP mmHg, mean (SD)** (n=24; n=13)		9.9 (3.8)	9.4 (3.5)	0.65

Comparison of baseline statistics between patients of the SAPH group where a variant in the diagnostic gene panel was identified (n=14) compared to patients without such a variant (n=25).

Supplemental Table S2 Baseline characteristics of SA patients with a variant in diagnostic gene panel compared to SA patients without a variant

		SA – no variant in diagnostic gene panel (n=34)	SA – with variant in diagnostic gene panel (n=5)	p-value
**Male, n (%)**		18 (52.9)	1 (20.0)	0.34
**Age mean (SD), y**		61.4 (11.1)	57.7 (9.6)	0.47
**Ancestry %**	White, n (%)	24 (70.6)	3 (60.0)	0.40
	Black, n (%)	8 (23.5)	1 (20.0)	
	Hindustan, n (%)	2 (5.9)	1 (20.0)	
	United States, n (%)	-	-	
**Scadding stage**	0, n (%)	7 (20.6)	1 (20.0)	1.00
	1, n (%)	3 (8.8)	0 (0.0)	
	2, n (%)	8 (23.5)	1 (20.0)	
	3, n (%)	5 (14.7)	1 (20.0)	
	4, n (%)	11 (32.4)	2 (40.0)	
**Lung fibrosis score**	< 5%, n (%)	19 (55.9)	3 (60.0)	0.89
	5-20%, n (%)	7 (20.6)	0 (0.0)	
	>20%, n (%)	8 (23.5)	2 (40.0)	
**FVC % predicted, mean (SD)** (n=31; n=5)		96.7 (21.8)	79.1 (27.7)	0.32
**DLCO SB% predicted, mean, mean (SD)** (n=28; n=4)		69.3 (15.9)	62.3 (7.7)	0.25

*Comparison of baseline statistics between subjects of the SA group where a variant in the diagnostic panel was identified (n=5) compared to subjects without such variant (n=34). Hemodynamics are not available for this group. *All subjects in this group were from the Dutch cohort*

Supplemental Table S3 Overview of rare variants in biologically relevant genes in which ≥15% of the SA patients carry a rare variant

**Gene**	**Transcript**	**Nucleotide change**	**Amino acid change**	**dbSNP**	**gnomAD frequency**	**CADD**	**SIFT**	**PPh2**	**Mutation Taster**	**Mutation Assessor**
PDE4A	NM_001111307.2	c.-129C>T		rs879405688	0.0004	18.4	-	-	B	-
PDE4A	NM_001111309.1	c.388G>A	p.(V130I)	rs1234243707	0.0000	23.3	D	PoD	B	M
PDE4A	NM_006202.3	c.487C>T	p.(R163W)	rs144901748	0.0001	28.6	D	PrD	D	M
PDE4A	NM_006202.3	c.748+1G>T			-	34.0	-	-	D	-
PDE4A	NM_006202.3	c.871C>T	p.(R291W)	rs146986676	0.0006	25.6	T	PoD	B	L
PDE4A	NM_006202.3	c.883C>T	p.(R295C)	rs951423867	-	31.0	D	PrD	B*	H
PDE4A	NM_006202.3	c.1018C>T	p.(R340C)	rs200220469	-	31.0	D	PrD	B**	H
PDE4A	NM_006202.3	c.1357G>A	p.(E453K)	rs139163380	0.0001	23.1	T	PoD	B	L

*a) SIFT: T= Tolerated; D = Damaging; A = Activating; b) PolyPhen-2: B= Benign; PrD = Probably Damaging; PoD = Possibly Damaging; c) Mutation Taster: D=Deleterious; B=Benign; d) Mutation Assessor functional impact: N= Neutral; L= Low; M= Medium; H= High; Predicted functional impact high or medium is predicted functional, low or neutral is predicted non-functional. Predictions by SIFT, PolyPhen-2, Mutation Taster or Mutation Assessor indicating deleterious consequences are underlined. * but predicted D in all other transcripts;** but predicted D in most other transcripts*
